# Single-Cell Transcriptome Sequencing and Proteomics Reveal Neonatal Ileum Dynamic Developmental Potentials

**DOI:** 10.1128/mSystems.00725-21

**Published:** 2021-09-21

**Authors:** Qingshi Meng, Liang Chen, Bohui Xiong, Beining Kang, Pengfei Zhang, Shanlong Tang, Hui Han, Wei Shen, Xiaohui Feng, Shengnan Feng, Ruqing Zhong, Xiangfang Tang, Sheng Zhang, Hongfu Zhang, Yong Zhao

**Affiliations:** a State Key Laboratory of Animal Nutrition, Institute of Animal Sciences, Chinese Academy of Agricultural Sciences, Beijing, People’s Republic of China; b College of Life Sciences, Qingdao Agricultural University, Qingdao, People’s Republic of China; c Proteomics and Metabolomics Facility, Cornell University, Ithaca, New York, USA; University of Maine

**Keywords:** ileum, neonatal development, scRNA-seq, proteomics, microbiota

## Abstract

The neonatal period is a crucial time during development of the mammalian small intestine. Moreover, neonatal development and maturation of the small intestine are exceptionally important for early growth, successful weaning, and postweaning growth and development, in order to achieve species-specific milestones. Although several publications recently characterized intestinal epithelial cell diversity at the single-cell level, it remains unclear how differentiation and molecular interactions take place between types and subtypes of epithelial cells during the neonatal period. A single-cell RNA sequencing (scRNA-seq) survey of 40,186 ileal epithelial cells and proteomics analysis of ileal samples at 6 time points in the swine neonatal period were performed. The results revealed previously unknown developmental changes: specific increases in undifferentiated cells, unique enterocyte differentiation, and time-dependent reduction in secretory cells. Moreover, we observed specific transcriptional factors, ligand-receptor pairs, G protein-coupled receptors, transforming growth factor β, bone morphogenetic protein signaling pathways, and gut mucosal microbiota playing vital roles in ileal development during the neonatal window. This work offers new comprehensive information regarding ileal development throughout the neonatal period. Reference to this data set may assist in the creation of novel interventions for inflammation-, metabolism-, and proliferation-related gut pathologies.

**IMPORTANCE** We found previously unknown neonatal ileum developmental potentials: specific increases in undifferentiated cells, unique enterocyte differentiation, and time dependent reduction in secretory cells. Specific transcriptional factors (TFs), ligand-receptor pairs, G protein-coupled receptors, transforming growth factor β, bone morphogenetic protein signaling pathways, and the gut mucosal microbiota are involved in this process. Our results may assist in the creation of novel interventions for inflammation-, metabolism-, and proliferation-related gut pathologies.

## INTRODUCTION

At birth, the neonatal mammalian intestine is similar in morphology to that of the adult; however, it is immature and undergoes rapid growth and development during the neonatal period (1–3). As the intestine grows, it digests and absorbs nutrients and interacts with the external milieu by secreting regulatory products to orchestrate whole-body development (1–3).

Pigs have been widely used in biomedical studies because pigs and humans have many common features in the physiology and microbiology of the gastrointestinal (GI) tract, and piglets are considered a promising model to investigate the GI tract development of humans (4, 5). Neonatal piglets are very suitable for in-depth studies of the GI development of human infant because of the unavailability of healthy human infant intestinal samples and impractically confounding variables in human studies (4, 5).

A number of recent publications characterize intestinal epithelial cellular diversity and the underlying mechanisms of development and differentiation at one point in time (adult or pathogenic stage) (2, 6) or during fetal development (7, 8) using single-cell RNA sequencing (scRNA-seq). However, it is still unknown how epithelial cell types and subtypes differentiate throughout the neonatal period and what the molecular interactions (ligand-receptor, transcriptional factors, etc.) of cell types or subtypes during this critical developmental window are.

We performed an scRNA-seq survey of ileal epithelial cells and proteomic analysis of ileal samples at 6 time points in the porcine neonatal window; identified different developmental potentials for different types of epithelial cells; explored the specific transcriptional factors, ligand-receptor pairs, G protein-coupled receptors (GPCRs), transforming growth factor β (TGF-β) signaling, and bone morphogenetic protein (BMP) signaling in different cell types during the neonatal period.

## RESULTS

### Dynamic development of the neonatal ileum epithelium from a single-cell survey and proteomic analysis.

**FIG 1:**
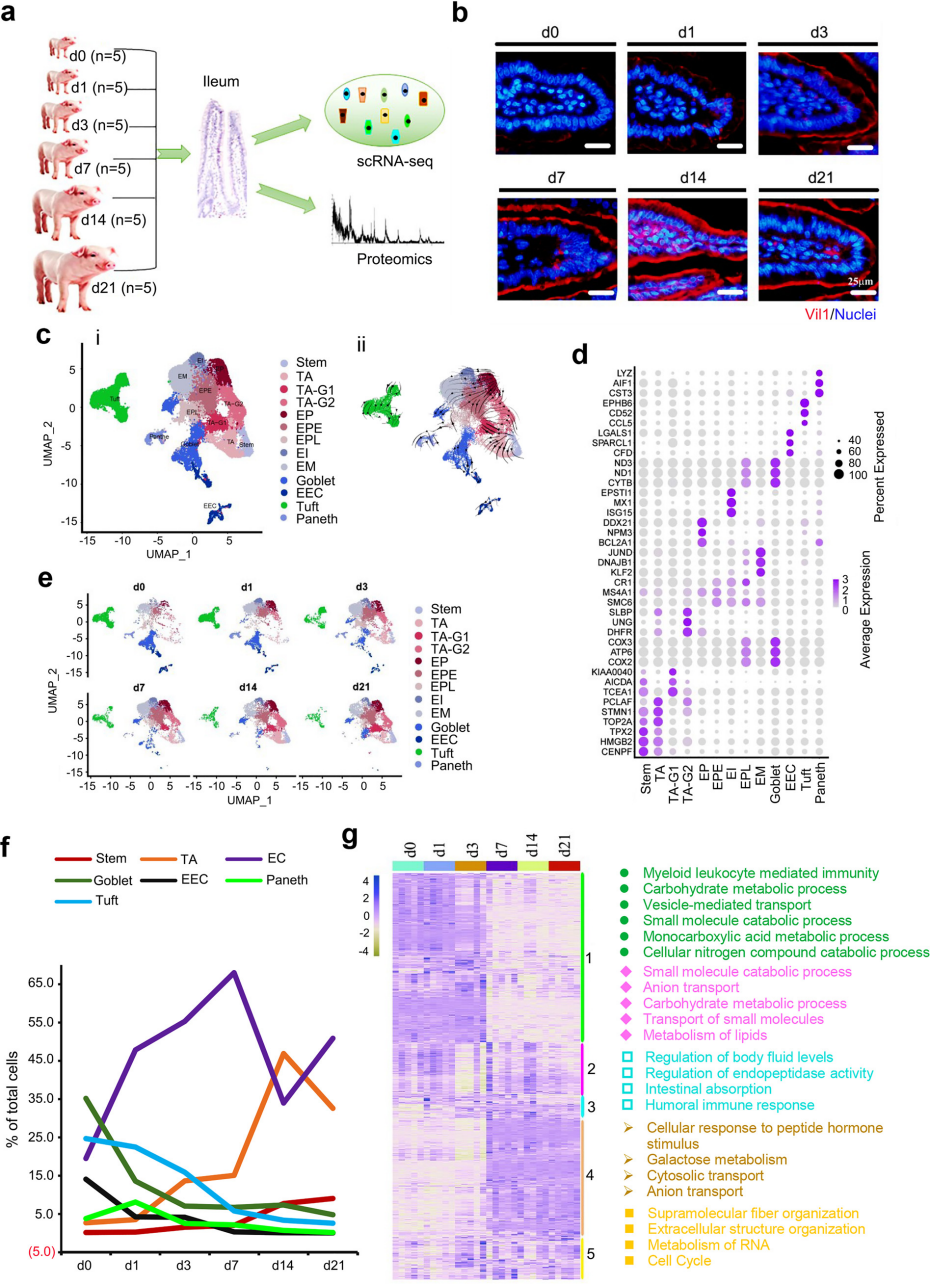
Single-cell survey of swine neonatal ileal epithelium. (a) Overview. (b) Vil1 staining of the ileum at 6 time points in ileal samples. (c) Cell type clusters. (Panel i) tSNE of 40,186 single cells (points), colored by cluster assignment (*n* = 5 piglets at each time point). (Panel ii) RNA velocity vectors projection on tSNE plot. (d) Heat map of cluster marker genes, colored by relative gene expression. Dot size represents the fraction of cells per cluster. (e) Cell population changes during development (from day 0 to day 21). (f) Proportion of cells in each cluster at each time point. (g) Proteomics data of piglet ileum at 6 time points with the enriched functions for each group of proteins.

In the current investigation, we explored piglet ileal development during the neonatal developmental window (birth [day 0] to 21 days of age [day 21]) using scRNA-seq and proteomics (Fig. 1a). The piglet ileum developed gradually during mucosal-layer maturation (Fig. 1b; Fig. S1a to d). There were 13 types (clusters) of cells with corresponding marker genes, including stem cells (SCs), transit-amplifying progenitors (TA), TA-G1, TA-G2, enterocyte progenitors (EP), early enterocyte progenitors (EPE), late enterocyte progenitors (EPL), immature enterocytes (EI), mature enterocytes (EM), enteroendocrine cells (EECs), and goblet, Paneth, and tuft cells (Fig. 1c and d; Fig. S1c and d). Developmental potential timing of cells was confirmed by RNA velocity analysis (Fig. 1c) (9, 10).

10.1128/mSystems.00725-21.1 FIG S1 Summary of single-cell RNA sequencing (scRNA-seq) data and data for undifferentiated cells (stem cells, TA, TA-G1, and TA-G2). (a) Histopathological images of swine ileum at different time points. (b) Summary of scRNA-seq for ileal tissue at different time points. (c) Heat map of the top 25 marker genes in each cluster of cells. (d) The expression pattern of marker genes in different clusters of cells. (e) Heat map of the top 25 marker genes in each cluster of undifferentiated cells. (f) Expression pattern of marker genes in different clusters of undifferentiated cells. (g) Trajectory reconstruction of undifferentiated cells based on cell clusters by pseudotime analysis. (i). Monocle plot. (ii). RNA velocity vectors projection on monocle plot. (h) Marker gene expression patterns in Monocle analysis. (i) Monocle images for different samples at 6 time points, or different clusters of undifferentiated cells. (j) Protein levels of HMGB1 in different samples at different time points by IHF. Download FIG S1, TIF file, 2.5 MB. Copyright © 2021 Meng et al. 2021 Meng et al. https://creativecommons.org/licenses/by/4.0/ This content is distributed under the terms of the Creative Commons Attribution 4.0 International license.

Ileal development during the neonatal window was reflected by the proportion of the different clusters of cells at different times (Fig. 1e and f). At day 0, goblet cells were most abundant, followed by tuft cells, enterocytes (EC; including EP, EPE, EPL, EI, and EM), EECs, Paneth cells, TA, and stem cells. However, with advancing age, ECs increased dramatically, peaking at day 7 and then dropping slightly at day 14 and day 21 (Fig. 1e and f). Meanwhile, stem cells and TA gradually increased (Fig. 1e and f). However, goblet cells and EECs were sharply reduced from day 0 to day 1 and continued to decrease until day 21. Tuft cells gradually dropped from day 0 to day 21, while Paneth cells increased slightly from day 0 to day 1 and then gradually decreased until day 21. At day 21, ECs were most common (50.87%), followed by TA (32.60%), stem cells (9.02%), goblet cells (4.78%), tuft cells (2.57%), Paneth cells (0.12%), and EECs (0.05%) (Fig. 1e and f). All cell types approached typical mature ileum development at day 21 (2, 8).

The 1,976 known proteins in swine ileal samples were clustered into 5 groups (Fig. 1g). Group 1 was the largest, including 747 proteins that were decreased during ileal development (Fig. 1g). These proteins were mainly associated with immune function and secretion, correlating the functions of goblet, Paneth, and tuft cells and EECs, which matched the scRNA-seq data (Fig. 1g). Protein levels in group 2 were similar from day 1 to day 21, with functions related to ECs (Fig. 1g). Group 3 proteins gradually increased during neonatal development, and their functions were also related to ECs (Fig. 1g). Group 4 protein levels were lower from day 0 to day 3 but higher from day 7 to day 21, and functions were related to ECs (Fig. 1g). Group 5 proteins were lower at day 0 and day 1 but then increased from day 3 to day 21; their functions were related to cell cycle (cell proliferation), similar to those of stem cells and TA (Fig. 1g). Overall, the proteomic data and scRNA-seq data matched well.

### Specific increase in undifferentiated cells.

**FIG 2:**
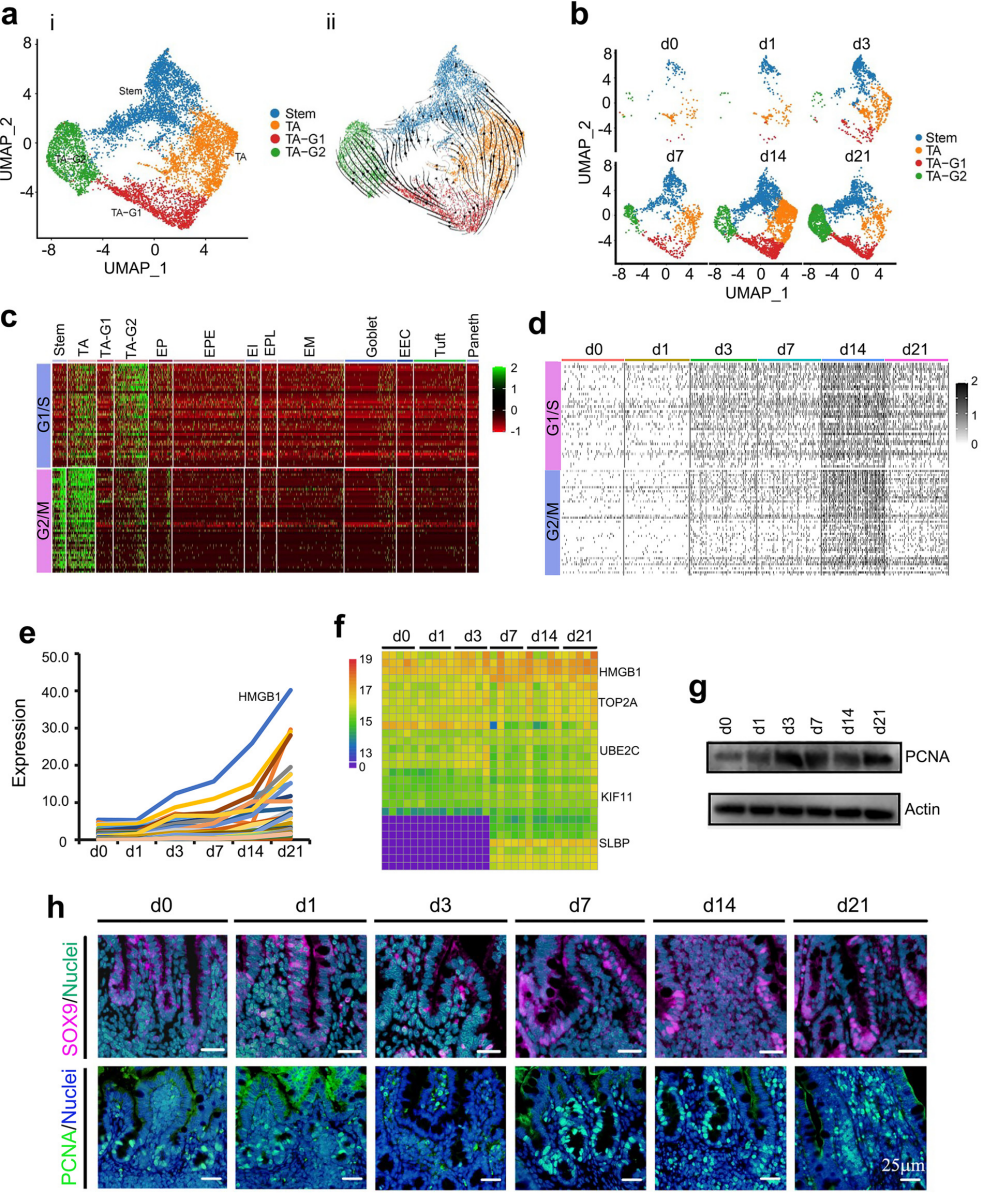
Increase in piglet ileum undifferentiated cells during the neonatal window. (a) Cell type clusters for undifferentiated cells. (Panel i) tSNE of undifferentiated single cells (points), colored by cluster assignment. (Panel ii) RNA velocity vectors projection on tSNE plot. (b) Undifferentiated cell population changes during development (from day 0 to day 21). (c) Heat map of cluster genes for the cell cycle in different clusters of cells. (d) Heat map of cluster genes for the cell cycle in the samples at different time points. (e) Expression pattern of the top 50 specifically expressed genes in undifferentiated cells. (f) Protein levels of the top 50 specifically expressed genes from the proteomic analysis. (g) Protein levels of PCNA in the different samples at different time points by WB. (h) Protein levels of PCNA in the different samples at different time points by IHF.

During neonatal development, the undifferentiated cells underwent specific and dramatic increases (Fig. 2a and b; Fig. S1e to j) from 2.82% to 41.62% during day 0 to day 21 (Fig. 1f and 2b). Unsupervised pseudotime, from stem cells to TA, TA-G1, and TA-G2, revealed the developmental trajectory of these undifferentiated cells (Fig. 2a; Fig. S1g to i, RNA velocity).

Cell cycle gene expression was high in the undifferentiated cells in comparison with EC or secretory cells (Fig. 2c) and was most profound at day 14, which matched the cell growth trend of these undifferentiated cells (Fig. 2d). To search the correlation of gene expression pattern and cell population, the expression levels of the top 50 specifically expressed genes from these undifferentiated cells were determined. The expression of most of these 50 genes gradually increased from day 0 to day 21 (Fig. 2e), which matched the increase in number of the undifferentiated cells. Moreover, the protein levels showed a similar trend to that of gene expression (Fig. 2f) in the proteomics data. PCNA protein levels increased from day 0 and day 1 to day 21 using Western blotting (WB) and immunohistofluorescence (IHF) analysis, which confirmed the proteomics data and scRNA-seq data (Fig. 2g and h). The protein level of stem cell marker SOX9 (11) elevated at day 7 to day 21 (Fig. 2h). Concurrently, the protein levels of another marker gene for undifferentiated cells, HMGB1, displayed a trend similar to that of PCNA (Fig. S1j).

### Unique maturation of enterocytes.

**FIG 3:**
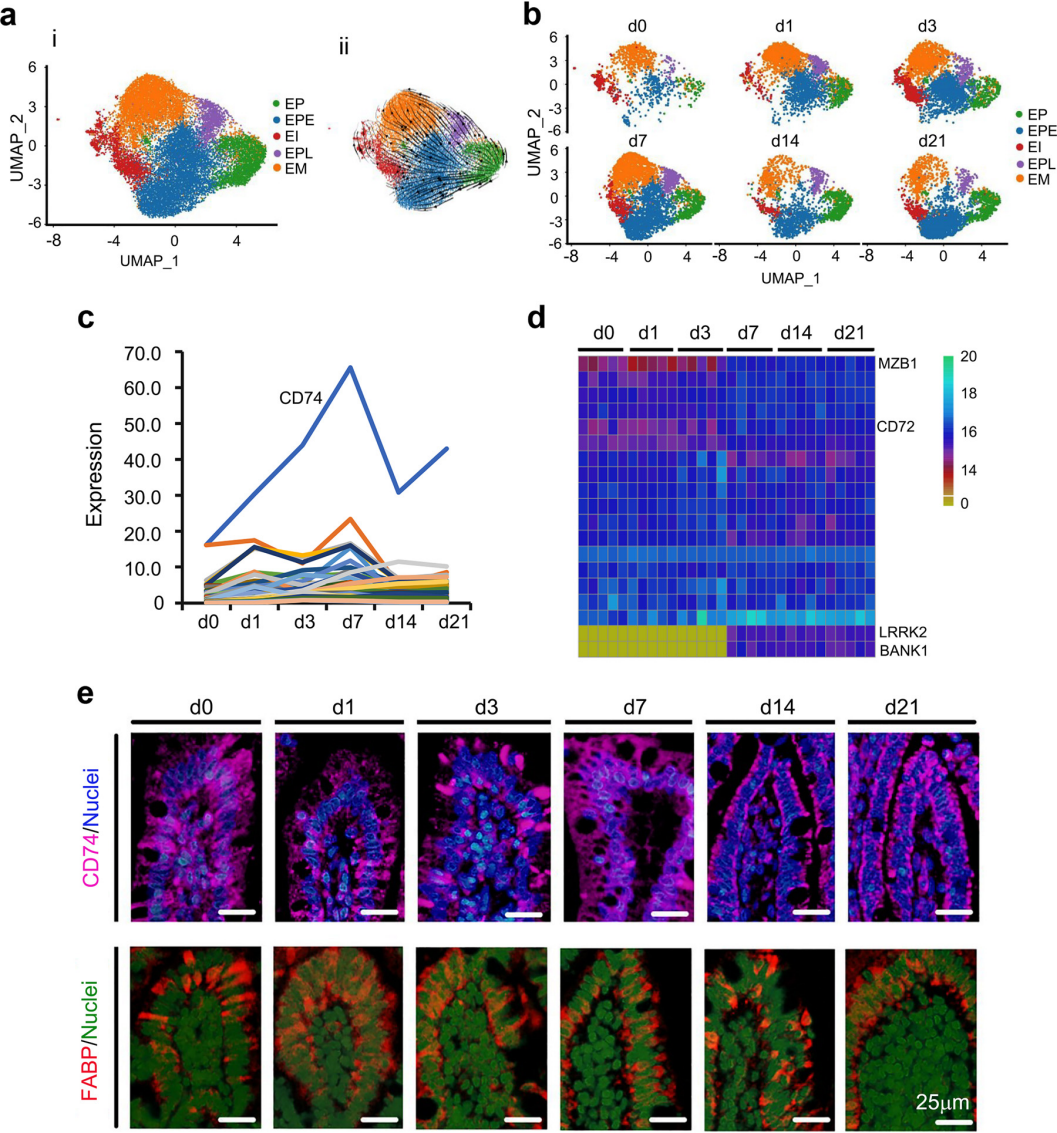
Differentiation of piglet ileal enterocytes during the neonatal window. (a) Cell type clusters for enterocytes (EP, EPE, EPL, EI, and EM). (Panel i) tSNE of enterocytes single cells (points), colored by cluster assignment. (Panel ii) RNA velocity vectors projection on tSNE plot. (b) Enterocyte cell population changes during development (from day 0 to day 21). (c) Expression pattern of the top 50 specifically expressed genes in enterocyte cells. (d) Protein levels of some of the top 50 specifically expressed genes from the proteomics analysis. (e) Protein levels of CD74 and FABP in the different samples at different time points by IHF.

In the mature small intestine, ECs (EP, EPE, EPL, EI, and EM) predominate, with few goblet, Paneth, and tuft cells and EECs (2, 12, 13). EC number quickly increased from day 0 to day 7 and then decreased at day 14 and day 21 (Fig. 1f and 3a and b; Fig. S2). Unsupervised pseudotime, from EP to EPE/EPL, EI, and EM, revealed the developmental trajectory of these ECs (Fig. 3b; Fig. S2c to e).

10.1128/mSystems.00725-21.2 FIG S2 Summary of scRNA-seq data for enterocytes. (a) Heat map of the top 25 marker genes in each cluster of enterocytes. (b) Expression pattern of marker genes in different clusters of enterocytes. (c) Trajectory reconstruction of enterocyte cells based on cell clusters following pseudotime analysis. (Panel i) Monocle plot. (Panel ii) RNA velocity vector projection on Monocle plots. (d) Marker gene expression patterns in Monocle analysis. (e) Monocle images for different samples at 6 time points, or different clusters of enterocytes. (f) Protein levels of catenin in different samples at different time points determined by IHF. Download FIG S2, TIF file, 2.5 MB. Copyright © 2021 Meng et al. 2021 Meng et al. https://creativecommons.org/licenses/by/4.0/ This content is distributed under the terms of the Creative Commons Attribution 4.0 International license.

To correlate gene expression patterns and cell population changes, the expression levels of the top 50 specifically expressed genes from ECs were analyzed. Their expression trends were similar to those in ECs during day 0 to day 21, especially CD74 (a survival receptor on intestinal epithelial cells) (14), whose trend was almost identical to that of ECs (Fig. 3c). Furthermore, the protein levels of some of these 50 genes from the proteomics analysis showed a trend similar to that of gene expression (Fig. 3d). The protein levels of CD74 followed the same trend as its gene expression pattern, which confirmed the proteomic data and scRNA-seq data (Fig. 3e). Another EC protein, FABP, was expressed in the piglet ileum (Fig. 3e) (2). The cell junction protein catenin was more condensed at day 7 to day 21 than that at day 0 to day 3, which indicated that the ileum matured with time (Fig. S2f).

### Time-dependent decrease in secretory cells.

There are 4 major types of secretory cells in the small intestine mucosal epithelium: goblet, Paneth, and tuft cells and EECs. All these types were present in the ileum from day 0 to day 21 (Fig. 1c to e) and showed a similar decreasing developmental trend (Fig. 1f).

**FIG 4:**
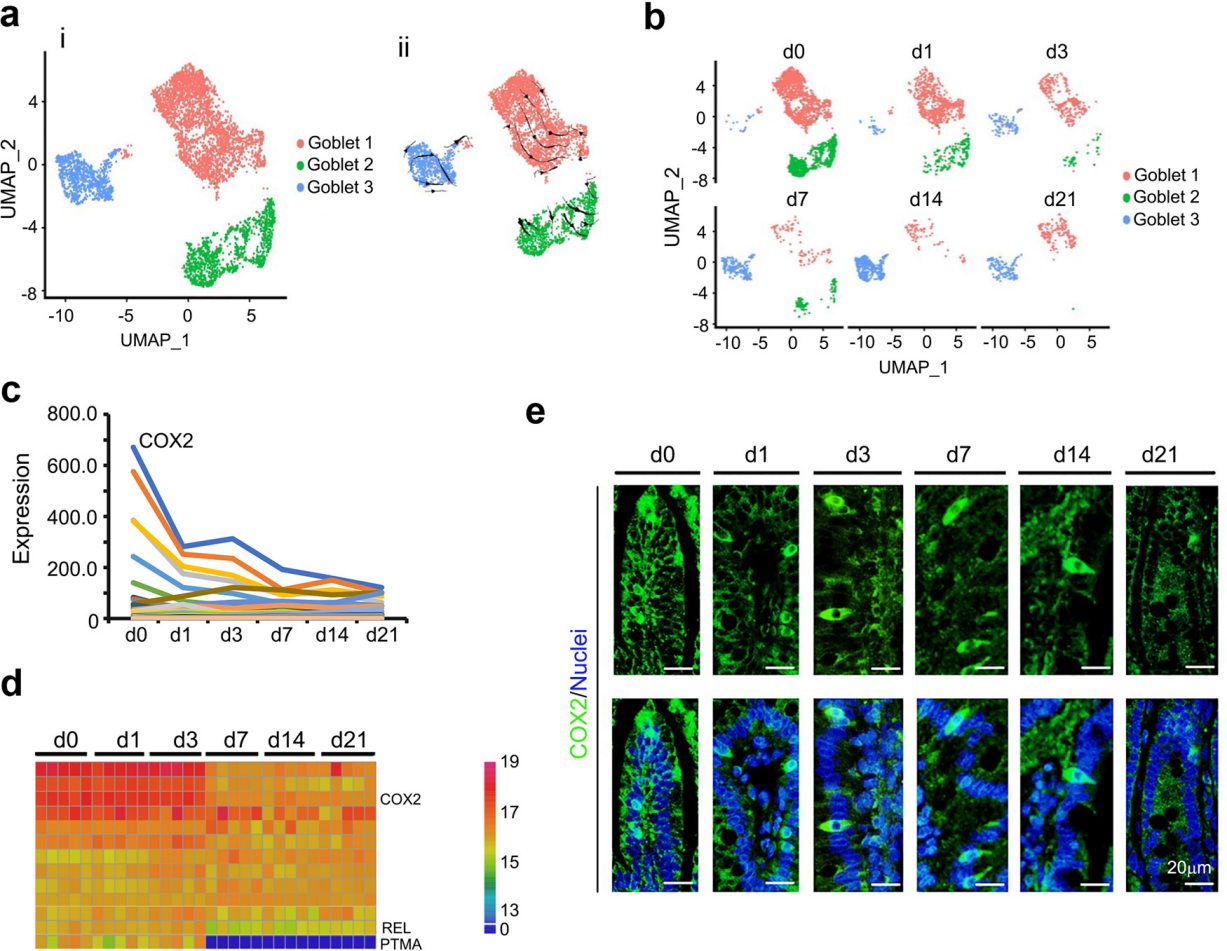
Decrease in goblet cells of piglet ileum during the neonatal window. (a) Cell type clusters for goblet cells. (i) tSNE of goblet single cells (points), colored by cluster assignment. (ii) RNA velocity vector projection on tSNE plot. (b) Decrease in goblet cell population during development (from day 0 to day 21). (c) Expression pattern of the top 50 specifically expressed genes in goblet cells. (d) Protein levels of some of the top 50 specifically expressed genes from the proteomics analysis. (e) Protein levels of COX2 in the different samples at different time points by IHF.

Goblet cells synthesize and secrete mucus (15) to assist with gut content elimination and immune defense (16). In the current investigation, goblet cells were most prolific at day 0, followed by ECs and tuft cells (Fig. 1f; Fig. S1a). Specifically, there were 3 clusters of goblet cells (Goblet 1, Goblet 2, and Goblet 3) (Fig. 4a; Fig. S3a and b). Although the total number of goblet cells continuously decreased (Fig. 1f and 4b), those of Goblet 3 cells gradually increased (Fig. 4b), which suggested that they may be more mature. Unsupervised pseudotime analysis and RNA velocity showed cell development sequencing from Goblet 1 to Goblet 2 to Goblet 3 (Fig. 4a; Fig. S3c to e). The top 50 specifically expressed goblet cell genes followed the same trend as the number of goblet cells, decreasing during day 0 to day 21, especially COX2, which was most highly expressed in goblet cells (Fig. 4c). Furthermore, the protein levels of some of these 50 genes followed the same trend as gene expression in goblet cells (Fig. 4d) in the proteomics analysis. COX2 protein levels followed the same trend as its gene expression pattern (Fig. 4d and e). At the same time, the goblet marker genes MUC13, TFF3, and ND3 were also present in goblet cells (Fig. S3f).

10.1128/mSystems.00725-21.3 FIG S3 Summary of scRNA-seq data for goblet cells and tuft cells. (a) The heat map of the top 25 marker genes in each cluster of goblet cells. (b) The expression pattern of marker genes in different clusters of goblet cells. (c) Trajectory reconstruction of goblet cells based on cell clusters following pseudotime analysis. (Panel i) Monocle plot. (Panel ii) RNA velocity vectors projection on monocle plot. (d) Expression pattern of marker genes in different clusters of goblet cells in monocle analysis. (e) Monocle images for different samples at 6 time points, or different clusters of goblet cells. (f) IHF for the protein expression of goblet cell marker genes MUC13, TFF3, and ND3. (g) Heat map of the top 25 marker genes in each cluster of tuft cells. (h) Trajectory reconstruction of goblet cells based on cell clusters following pseudotime analysis. (Panel i) Monocle plot. (Panel ii) RNA velocity vectors projection on monocle plot. (Panel iii) The expression pattern of marker genes in different clusters of tuft cells in monocle analysis. (i) Expression pattern of marker genes in different clusters of goblet cells in monocle analysis. (j) Monocle images for different samples at 6 time points, or different clusters of tuft cells. (k) IHF for the protein expression of IL-6 in samples from different time points. Download FIG S3, TIF file, 1.9 MB. Copyright © 2021 Meng et al. 2021 Meng et al. https://creativecommons.org/licenses/by/4.0/ This content is distributed under the terms of the Creative Commons Attribution 4.0 International license.

**FIG 5:**
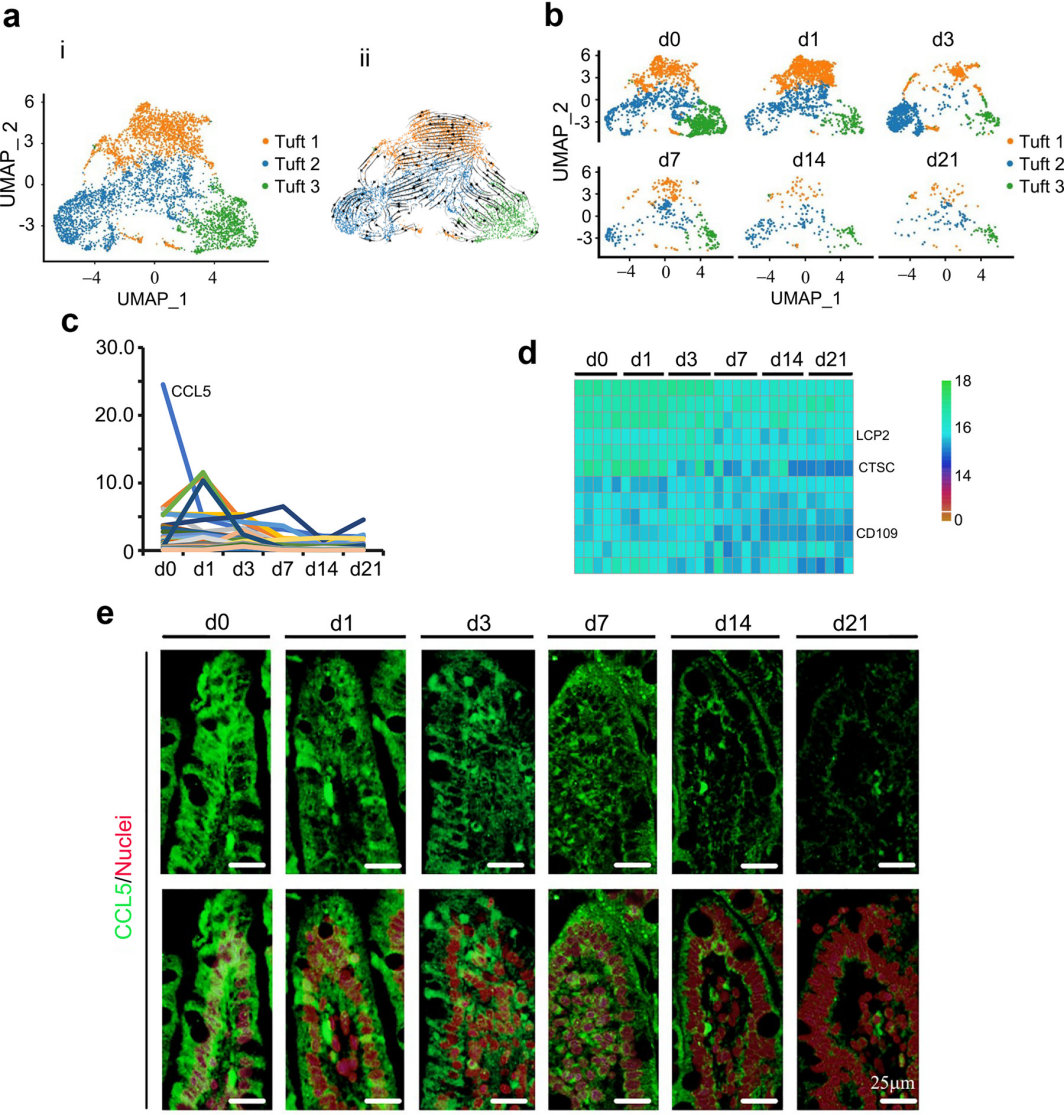
Reduction in tuft cells of piglet ileum during the neonatal window. (a) Cell type clusters for tuft cells. (i) tSNE of tuft single cells (points), colored by cluster assignment. (ii) RNA velocity vectors projection on tSNE plot. (b) Tuft cell population reduction during development (from day 0 to day 21). (c) Expression pattern of the top 50 specifically expressed genes in tuft cells. (d) Protein levels of some of the top 50 specifically expressed genes from the proteomics analysis. (e) Protein levels of CCL5 in the different samples at different time points by IHF.

In the current investigation, tuft cells were the second largest population of epithelial cells at day 0, and then decreased gradually from day 1 to day 21 (Fig. 1f and 5a and b). There were 3 subclusters of tuft cells (Tuft 1, Tuft 2, and Tuft 3), with Tuft 1 (Fig. 5a and b; Fig. S3g to k) being close to the progenitors as they developed into Tuft 2 and/or Tuft 3 (Fig. 5a; Fig. S3h, RNA velocity). The number of Tuft 1 cells increased from day 0 to day 1 followed by a decrease until day 21, while the number of Tuft 2 and Tuft 3 cells continued decreasing from day 0 to day 21 (Fig. 5b). The expression levels of the top 50 specifically expressed tuft cell genes followed the trend of tuft cells, decreasing from day 0 to day 21, especially the most highly expressed gene, CCL5 (Fig. 5c). Furthermore, the protein levels of some of these 50 genes followed the trend of gene expression in tuft cells (Fig. 5d) in the proteomics analysis. CCL5 protein levels followed the trend of gene expression, which confirmed the proteomic data and scRNA-seq data (Fig. 5e). The protein levels of another cytokine, interleukin 6 (IL-6), decreased during this time (Fig. S3k).

In the current investigation, EECs in the neonatal piglet ileal epithelium were classed into 5 subclusters with respective marker genes (Fig. S4). Sox4 was expressed in all 5 EEC subclusters, which suggested that these cells may have been at the progenitor stage, as EECs are reported to possess intestinal stem cell activity (17). The number of EECs continued dropping from day 0 to day 21 (Fig. S4b), and the expression levels of the top 50 specifically expressed genes in EECs followed the same trend (Fig. S4f). Furthermore, the protein levels of some of these 50 genes followed the trend of gene expression in EECs (Fig. S4g) in the proteomics analysis. The protein levels of CHGA (marker gene of EECs) followed its gene expression pattern, which confirmed the proteomic data and scRNA-seq data (Fig. S4h).

10.1128/mSystems.00725-21.4 FIG S4 Summary of scRNA-seq data for EECs and Paneth cells. (a) Cell type clusters for EECs. (Panel i) tSNE of goblet single cells (points), colored by cluster assignment. (Panel ii) RNA velocity vectors projection on tSNE plot. (b) EEC population decrease during development (from day 0 to day 21). (c) Heat map of the top 25 marker genes in each cluster of EECs. (d) Expression pattern of marker genes in different EEC clusters. (e) EEC trajectory reconstruction based on cell clusters following pseudotime analysis. (Panel i) Monocle plot. (Panel ii) RNA velocity vectors projection on monocle plot. (f) Expression pattern of the top 50 specifically expressed genes in EECs. (g) Protein levels of some of the top 50 specific expressed genes from the proteomic analysis. (h) Protein levels of CHGA in the different samples at different time points by IHF. (i) Cell type clusters for Paneth cells. (Panel i) tSNE of goblet single cells (points), colored by cluster assignment. (Panel ii) RNA velocity vectors projection on tSNE plot. (j) Paneth cell population decrease during development (from day 0 to day 21). (k) Heat map of the top 25 marker genes in each cluster of Paneth cells. (l) Expression pattern of marker genes in different clusters of Paneth cells. (m) Trajectory reconstruction of Paneth cells based on cell clusters following pseudotime analysis. (Panel i) Monocle plot. (Panel ii) RNA velocity vectors projection on monocle plot. (n) Expression pattern of the top 50 specifically expressed genes in Paneth cells. (o) Protein levels of some of the top 50 specifically expressed genes from the proteomic analysis. (p) Protein levels of LYZL1 in the different samples at different time points by IHF. Download FIG S4, TIF file, 1.7 MB. Copyright © 2021 Meng et al. 2021 Meng et al. https://creativecommons.org/licenses/by/4.0/ This content is distributed under the terms of the Creative Commons Attribution 4.0 International license.

Paneth cells, located at the base of crypts, are highly specialized with intensive secretory activity due to their extensive endoplasmic reticulum and Golgi network structures (18, 19). Paneth cells possess important antimicrobial functions in the small intestine because their large granules can release antimicrobial molecules, including peptides (18, 19). In the current study, there were two subclusters of Paneth cells with different marker genes (Paneth 1 and Paneth 2) (Fig. S4i to p). Overall, the number of Paneth cells gradually dropped from day 0 to day 21 (Fig. S4j). The expression levels of the top 50 specifically expressed genes in Paneth cells followed the cell number trend, decreasing from day 0 to day 21, especially for FTH1 and B2M (Fig. S4n). Furthermore, the protein levels of some of these 50 genes followed the trend of Paneth cell gene expression, especially for B2M (Fig. S4o) in the proteomics analysis. Protein levels of the Paneth cell marker gene LYZ (2) followed the trend of its gene expression pattern, which confirmed the proteomics data and scRNA-seq data (Fig. S4p).

Birth marks the transition from a sterile uterine environment to a microbe-rich environment (1, 20–23). The intestinal microbiota plays important roles in shaping intestinal epithelial development (1, 20–23). At birth, almost no microbiota was found in piglet ileal mucosa, while with development, the diversity of ileal microbiota increased (Fig. S5a to e). The relative proportion of the 4 major microbes changed during the neonatal window (Fig. S5d). As we found in our previous study (24), the “beneficial” microbiota member *Lactobacillus* started to appear in the swine ileum at day 3 and remained at a constant level until day 21, while the other 3 major microbiota organisms either decreased or fluctuated during this developmental window. This may be because the experimental piglets were raised solely on maternal milk, without antibiotics, immunization, or other additives, which was reflected by the proportion of the 4 secretory and protective cell types that decreased from day 0 to day 21 (Fig. 1e, 4b, and 5b; Fig. S4b and j). Correlations between microbiotas and different cell type development showed a positive correlation of lactobacillus with stem cells, TA, and ECs but a negative correlation with goblet, Paneth, and tuft cells and EECs (Fig. S5e).

10.1128/mSystems.00725-21.5 FIG S5 Changes in ileal mucosa microbiota and expression patterns of GPCRs and members of the TGF-β and BMP signaling pathways. (a) Alpha index of the small intestine microbiota: Shannon, Simpson, Chao1, and ACE indexes. (b) PLS-DA of the microflora in samples from different time points. (c) Differences of bacterial abundance at the genus level. (d) Relative proportion of the 4 major microbiotas. (e) Pearson correlation of the proportion of microbiotas and the proportion of different clusters of cells. (f) IHF images of some of the TFs at different time points from day 0 to day 21. (g) Expression pattern of GPCRs in different clusters of the ileal epithelium. (h) Expression pattern of GPCRs in samples from different time points. (i) Expression patterns of members of TGF-β and BMP pathways in different clusters of the ileal epithelium. (j) Expression patterns of members of TGF-β and BMP pathways in samples from different time points. Download FIG S5, TIF file, 2.0 MB. Copyright © 2021 Meng et al. 2021 Meng et al. https://creativecommons.org/licenses/by/4.0/ This content is distributed under the terms of the Creative Commons Attribution 4.0 International license.

### Active interactions of the different types of ileal epithelial cells during development.

**FIG 6:**
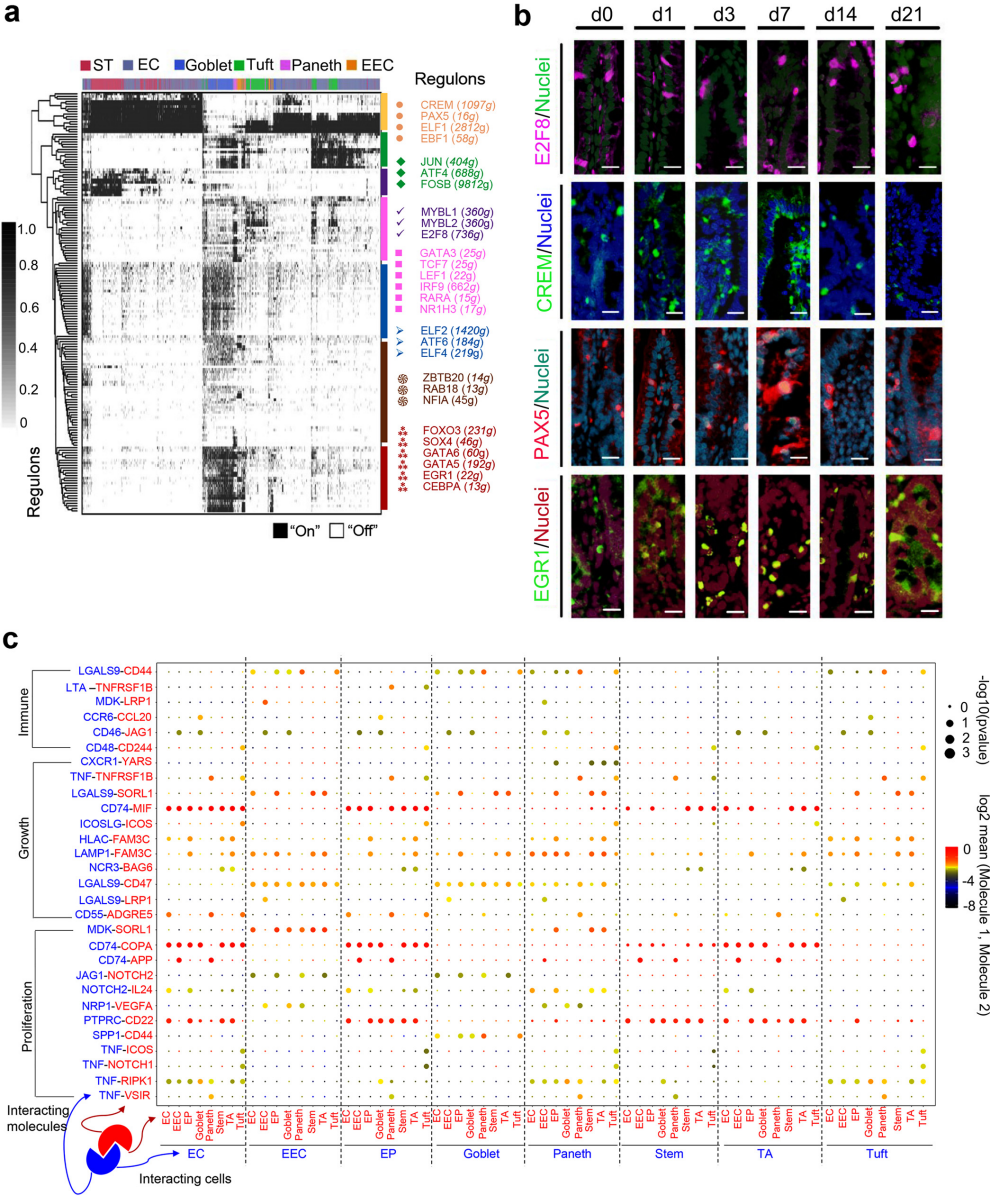
Multiple regulatory networks in ileal epithelial cells in the neonatal window. (a) SCENIC binary regulon activity heat map depicting different clusters of ileal epithelium cell enriched regulons. Column depict single cells, while rows depict regulons. “On” indicates active regulons, while “Off” indicates inactive regulons. (b) IHF images of some of the TFs at different time points from day 0 to day 21. (c) Overview of selected ligand-receptor interactions. *P* values are indicated by circle size (scale on right) (permutation test; see Materials and Methods). The means of the average expression level of interacting molecule 1 in cluster 1 and interacting molecule 2 in cluster 2 are indicated by color. Assays were performed at the mRNA level, but are extrapolated to protein interactions.

The analysis of gene regulatory networks (GRNs; transcriptional factors [TFs]) (25) among the different ileal epithelia revealed the presence of many master regulators within each cell population (Fig. 6a). Notably, the binary regulon activity heat map indicated that stem cells plus TA (ST) and EC predominantly posed a high expression of regulons, while the four secretary cell clusters had relatively low regulon expression (Fig. 6a). TF protein levels confirmed their gene expression (Fig. 6b; Fig. S5f), including CREM, E2M8, PAX5, EGR1, RAB18, and Pou2AF1.

Ligand-receptor complexes are the major form of cell-cell communication; this is critical for coordinating various biological activities, including development, differentiation, etc. (26, 27). To systematically explore cell type interaction in the ileum epithelial interface, we used CellPhoneDB analysis (www.CellPhoneDB.org) to explore ligand-receptor interacting pairs (Fig. 6c). Overall, ligand-receptor interaction was higher in ECs (EI and EM), EP (EP, EPE, and EPL), stem cells, and TA (TA, TA-G1, and TA-G2) with each other or with secretory cells (goblet, Paneth, and tuft cells and EECs) compared with the interaction of goblet, Paneth, and tuft cells and EECs with each other or with EC, EP, stem cells, and TA (Fig. 6c). The more significant pairs were CD74-MIF, CD74-COPA, PTPRC-CD22, and CD74-APP, which were mainly in the interaction of EC, EP, stem cells, and TA with each other or with secretory cells. The significant pairs in secretory cells were MDK-SORL1, LGALSL9-CD47, LAMP1-FAM3C, HLAC-FAM3C, and others. Some of these pairs have been reported to have broad functions; for example, CD74-MIF is involved in many biological processes in cardiac function, tumor formation, and inflammation modulation (28).

GPCRs, TGF-β signaling, and BMP signaling play important roles in small-intestinal development (2, 7). In total, 134 GPCRs were expressed in the ileal epithelium. Some of these receptors, such as SSR4 (signal sequence receptor subunit 4), SSR3, SSR2, and SSR1, were specifically expressed in some cell types or at some time points (Fig. S5g and h). The TGF-β signaling pathway members TGFBR2, TGFBR1, SMAD7, SMAD4, and SMAD2 and the BMP signaling pathway members BMP2K and BMP4 were also specifically expressed in some cell types or at some time points (Fig. S5i and j). The data indicated that these factors may be involved in ileal epithelial development during the neonatal window.

## DISCUSSION

We have provided the first large-scale scRNA-seq and proteomics study of the swine ileum at 6 time points during the neonatal window to delineate the developmental potential of different cell types. At birth, the piglet GI tract is relatively immature and is dependent on maternal milk for development and maturation (3). However, the relative weight of neonatal swine small intestines increases 30% to 40% by day 1; this fast growth results in a maximum length in the second week of life (29–32). Meanwhile, enterocyte number increased sharply from day 0 to day 1 and then continued to increase to a maximum at day 7. The number of TA (TA, TA-G1, and TA-G2) gradually increased from day 0 to day 14 and then dropped a little at day 21. The proteomics data matched the scRNA-seq data well to support ileal developmental potential in the neonatal period.

Tuft cells (brush or caveolated cells) (33) are chemosensory and play important roles in gut immune responses (34, 35). Few tuft cells have been reported in adult intestines; however, there are no reports of tuft cells in neonatal humans at the single-cell level. In the neonatal piglet ileum, tuft cells were a large population at day 0 and then decreased. Intestinal EECs are now regarded as key sensory cells (36, 37) secreting various hormones and playing vital roles in nutrient and microbial product metabolism (38, 39). EECs consist of many overlapping subclusters (36, 39). Cells that express Sct, Cck, Gcg, or GIP are typically called S, I, L, and K cells (36). Haber et al. classified EECs into 12 subclasses in adult mouse small intestinal epithelium with the specific marker genes: SOX4, Neurod1, Neurog3, Sct, CCK, Gcg, Pyy, Ghrl, Tac, Tph1, etc. (2). We found many common marker genes (SOX4, Neurod1, Pyy, Ghrl, Tac, and Tph1) as reported by Haber et al. (2); however, some marker genes were not found in piglet samples (Reg4, Neurog3, Sct, Cck, Gcg, Gal, Gip, Sst, and Nts).

Maternal milk, especially colostrum, is rich in bioactive substances, immunoglobulins, and relatively large protein particles that are critical for intestinal and even whole organism development (29, 40, 41). Our study revealed the ileal development under natural conditions at a single-cell level.

It is known that cell type-specific TFs, GPCRs, and members of the TGF-β and BMP signaling pathways play important roles in small-intestinal epithelial cell development during the fetal stage or in response to pathogens (2, 5). Intestinal stem cells (ISC) play vital roles in intestinal epithelium renewal, and they generated a pool of highly proliferative TA cells; all these cells formed the undifferentiated cell pool, with the potential to develop into all types of mature cells: EC, Paneth, goblet, and tuft cells and EECs (2, 4, 8). We found that, during the neonatal period, cell type differentiation was regulated by cell-intrinsic changes to regulatory programs: ligand-receptor pairs and the listed factors. The ligand-receptor complexes reflect cell-cell communication, which is crucial for a diverse range of biological processes, including development, differentiation, and inflammation (25, 26). We found a few important ligand-receptor pairs, such as CD74-MIF, CD74-COPA, PTPRC-CD22, and CD74-APP, with broad biological functions (14, 28). Novel interventions may be achieved to manage the inflammation-, metabolism-, and proliferation-related gut pathologies based on this work.

## MATERIALS AND METHODS

### Piglets.

All animal procedures used in this study were approved by the Animal Care and Use Committee of the Institute of Animal Sciences of Chinese Academy of Agricultural Sciences. Thirty full-term-born piglets of three pure-line large white sows were used in this investigation. All the piglets were kept in the same heat-preserving pigsty at 28°C and fed with maternal milk (no antibiotics, immunizations, or additives). Ileum mucosa was collected from 5 piglets at day 0 (at birth; *n* = 5), day 1 (1 day after birth; *n* = 5), day 3 (3 days after birth; *n* = 5), day 7 (7 days after birth; *n* = 5), day 14 (14 days after birth; *n* = 5), and day 21 (21 days after birth; *n* = 5). At each time point, part of the ileum tissue was removed and fixed in 10% formaldehyde to make the blocks for histochemical analysis. Part of the ileum mucosa was gently scraped after washing with phosphate-buffered saline (PBS) buffer three times; then the tissue was used for the isolation of single cells for scRNA-seq analysis. Part of the ileum mucosa was gently scraped after PBS buffer washing three times, quickly frozen with liquid nitrogen, and then kept in a −80°C refrigerator for proteomics and Western blotting (8, 24).

### Single-ileal-cell isolation, library preparation, sequencing, and data analysis.

**(i) Single-cell isolation, library preparation, and sequencing.** Single-cell libraries were constructed using a 10× Genomics chromium single cell 3′ library and gel bead kit v.2 (10× Genomics Inc., Pleasanton, CA, USA; 120237) according to the manufacturer’s instructions. The protocols for single-cell sample preparation, library construction, and sequencing are recorded in our previous report (8) and that of Haber et al. (2). In summary, piglet ileal sections were collected and washed with PBS. Subsequently, tissues were incubated in 20 mM EDTA-PBS for 90 min on ice, being agitated every 30 min. Following a 90-min incubation, the samples underwent vigorous agitation and the supernatant was removed and placed in a new tube. All samples were then incubated with fresh 20 mM EDTA-PBS on ice for 30 min, and the supernatant was collected again. In total, four separate fractions were collected and then mixed. Following centrifugation at 300 × *g* for 3 min, each cell pellet was collected and washed twice with PBS using the same centrifugation program. Cells were then digested with TrypLE Express (Invitrogen) for 1 min at 37°C and single cells were collected using a 40-mm filter. Cells were again washed twice, this time with PBS solution supplemented with 0.04% bovine serum albumin (BSA; Sigma, St. Louis, MO, USA; A1933). Trypan blue staining and a hemocytometer (Bio-Rad, Hercules, CA, USA; TC20) were used to examine cell viability (>95%). A concentration of 1,000 cells/μl was created for loading onto the single cell chip (one/group). A chromium 10× single-cell system (10× Genomics) was used in the Gel Bead in EMulsions (GEMs) system. Subsequently, cells were barcoded, and a cDNA library was constructed. Sequencing was performed using an Illumina Novaseq 6000 sequencer (Illumina, San Diego, CA, USA) with paired-end 150-bp (PE150) reads.

**(ii) Single-sample analysis and aggregation.** CellRanger software (10× Genomics) was applied to process the data sets with the “–force-cells = 5000” argument. The porcine reference genome (https://www.ncbi.nlm.nih.gov/assembly/GCF_000003025.6/) was used and was built with the “cellranger mkgtf” function. After the CellRanger analysis, the gene barcode matrices were processed with the Seurat single-cell RNA-seq analysis R package in Rstudio (v3.0) (42). Cells with fewer than 200 minimal genes and genes expressed in fewer than 3 cells were removed to maintain high-quality data sets for downstream analysis. After normalization, the three data sets (one from each of the three treatment groups) were merged using the Seurat RunMultiCCA function. Characterized cell clusters were reviewed using the Seurat RunTSNE function based on the t-distributed Stochastic Neighbor Embedding (tSNE) algorithm and default settings. The FindClusters function was used to calculate cell clusters, and cell cluster markers were found using the Seurat FindAllMarkers function.

**(iii) Subclustering and Gene Ontology enrichment analysis.** When all cell clusters in the piglet ileal samples had been characterized, cells were further clustered according to cell identity. To obtain similar cell types for downstream analysis, the SubsetData function was used. When clustering was complete, cluster-specific marker genes were identified using the FindAllMarkers function. The marker genes were used by Metascape (http://metascape.org) for enrichment analysis.

**(iv) Single-cell pseudo-time trajectory analysis.** A single-cell pseudo-time trajectory (http://cole-trapnell-lab.github.io/monocle-release/tutorials/) (43, 44) was determined using Monocle 2. The Monocle object was formed using the Monocle-implemented newCellDataSet function from the Seurat object with a lower detection limit of 0.5. Seurat was used to identify variable genes for ordering. Dimensionality was constructed using the DDRTree method with regression of the number of unique molecular identifiers (UMIs). Root state was collected following their Seurat cell identity information, and branch-specific gene expression was calculated using the Monocle-implemented BEAM function. The branched heat map was further constructed using the “plot_genes_branched_heat map” function.

**(v) Single-cell regulatory-network analysis.** In order to identify gene regulatory networks that are active during ileal cell development, we performed regulatory network inference and clustering using SCENIC (https://github.com/aertslab/SCENIC); this is a modified method of inferring gene regulatory networks from single-cell RNA-seq data (8, 25). During analysis for the single-cell RNA-seq expression matrix, cell IDs were placed in columns and genes were placed in rows. Then, the geneFiltering function was used to remove genes with UMI counts across all samples of <100 and those that were expressed in <1% of cells. GENIE3 was then used to infer coexpression matrix containing potential regulators. RcisTarget was used to identify potential direct-binding targets based on DNA motif, analysis and we used databases (mm 10) that scored the motifs in the promoter of the genes (up to 500 bp upstream of the transcription start site [TSS]) and in the 10 kb around the TSS (±10 kb). The AUCell algorithm was used to calculate regulon activity in each cell and to convert the network activity into ON/OFF (binary activity matrix) with default settings.

**(vi) RNA velocity analysis by velocyto.** RNA velocity using the earlier described velocyto package (9, 10) was used to establish whether a differentiation relationship was present in neonatal ileal cells. Using the standard protocol, counts of unspliced and spliced mRNA in piglet ileal cells were generated using the velocyto CLI. RNA velocity was then determined in all types of ileal cells (“all”) or specific ileal cell types using a similar workflow and parameters. Subsequently, RNA velocity was calculated under the assumption of constant velocity and transition probability, and embedding shift was calculated based on the previously generated UMAP representation of the ileum data set.

**(vii) Protein-protein network (ligand-receptor) enrichment analysis (26, 27).** CellPhoneDB analysis (using the CellPhoneDB Python package [1.1.0]) was used to investigate the way in which context-dependent cross talk of different cell types enabled physiological processes to proceed; CellPhoneDB is a publicly available repository of curated receptors, ligands, and their interactions. Single-cell data from all types of cells were input into CellPhoneDB for cell-cell interaction analysis. Enriched receptor-ligand interactions between two cell types were derived based on the expression of a receptor by one cell type and the expression of the corresponding ligand by another cell type. Then, we identified the most relevant cell type-specific interactions between ligands and receptors, and only receptors and ligands expressed in more than 10% of the cells in the corresponding subclusters were considered. Pairwise comparisons were performed between the included cell types. We first randomly permuted the cluster labels of all cells 1,000 times to determine the mean of the average receptor and ligand expression levels of the interacting clusters. This generated a null distribution for each receptor-ligand pair. By calculating the proportion of the means that were higher than the actual mean, a *P* value for the likelihood of the cell type specificity of the corresponding receptor-ligand complex was obtained. We then selected interactions that were biologically relevant.

In summary, we profiled an scRNA-seq survey of ileal epithelia: 10,888 individual cells at day 0, 12,681 cells at day 1, 10,643 cells at day 3, 15,185 cells at day 7, 17,289 cells at day 14, and 15,492 cells at day 21. After quality control, 40,186 cells (6,487, 6,528, 6,762, 6,617, 6,949, and 6,643 cells for day 0, day 1, day 3, day 7, day 14, and day 21, respectively) were combined for further analysis. The cells were partitioned into 13 groups using unsupervised graph clustering (visualized by t-stochastic neighborhood embedding [tSNE]) as reported in previous studies (2, 8). Each cluster was characterized by a distinct cell type (2, 8): stem cells (SCs), transit-amplifying progenitors (TA), TA-G1, TA-G2, enterocyte progenitors (EP), early enterocyte progenitors (EPE), late enterocyte progenitors (EPL), immature enterocytes (EI), mature enterocytes (EM), enteroendocrine cells (EEC), and goblet, Paneth, and tuft cells with corresponding marker genes.

### Proteomics analysis.

Ileal sample proteomics analysis was performed as reported in our earlier publications (45, 46).

**(i) Protein extraction and digestion.** Ileal mucosa was homogenized in lysis buffer (100 mM Tris-HCl [pH 8.5], 7 M urea, 1% SDS, 5 mM TCEP [Tris(2-carboxyethyl)phosphine hydrochloride], protease inhibitors cocktail) at room temperature (RT). The bicinchoninic assay (BCA) was used to determine protein concentration, where 50 μg of protein was reduced with 5 mM TCEP at 56°C for 30 min and subsequently alkylated with 20 mM iodoacetamide in the dark, at RT for 30 min. Proteins were then precipitated using methanol-chloroform. Briefly, 4, 1, and 3 volumes of methanol, chloroform, and water, respectively, were added to the lysate; vortexing was performed after the addition of each solvent; and a final centrifugation took place in RT at 5,000 × *g* for 5 min. The supernatant was removed, cold methanol was used to wash the precipitate, and the sample was air dried. Finally, the precipitate was resuspended in 100 μl of digestion buffer (100 mM triethylammonium bicarbonate [TEAB] buffer; pH 8.0), trypsin was added at 1:25 (wt/wt), and protein digestion took place overnight at 37°C.

**(ii) TMTpro labeling.** Two sets of TMTpro Plex amine-reactive reagents were used to label 30 samples (46). Channel 126 was used to label an equally proportioned sample as the reference channel. Briefly, the reactive reagents were resuspended in 30 μl of anhydrous acetonitrile; they were added to each sample and mixed by vortexing. Reactions proceeded at room temperature for 1 h and were halted by the addition of 8 μl of 5% hydroxylamine for 15 min. The labeled samples were then pooled, freeze-dried, and resuspended in 20 μl of 0.1% formic acid and 2% acetonitrile in water for a fraction. Peptides were then loaded onto a Waters XBridge C_18_ column (5 μm, 4.6 by 100 mm, 120 Å). Ammonium formate in water formed buffer A (10 mM; pH 10) and ammonium formate in acetonitrile formed buffer B (10 mM; pH 10). Peptides were separated according to the following gradients: 0 to 3 min, 5% B; 3 to 40 min, 60% B; 40 to 48 min, 80% B; 48 to 52 min, 80% B; 52 to 53 min, 5% B; and 53 to 55 min, 5% B. In total, 44 fractions were collected, dried in a SpeedVac, blended into 11 fractions, and resuspended in 0.1% formic acid and 2% acetonitrile for subsequent nano-liquid chromatography–tandem mass spectrometry (nano-LC–MS/MS) analysis.

**(iii) LC-MS/MS.** Nano-LC–MS/MS analysis was performed using an Orbitrap Fusion Tribrid MS (Thermo Scientific, San Jose, CA, USA) equipped with a nanospray flex ion source and coupled with a Dionex UltiMate 3000 RSLC nano system (Thermo, Sunnyvale, CA, USA). Peptide samples (2 μl) were injected into the PepMap C_18_ columns (75 μm by 3 mm, 3 μm) at 6 μl/min for on-line enrichment and then separated on a PepMap C_18_ column (2 μm, 75 μm by 250 mm), using 0.1% formic acid as buffer A and 0.1% formic acid in 80% acetonitrile as buffer B at 300 nl/min. The peptides were eluted using the followed gradients: 0 to 5 min, 5 to 12% B; 5 to 65 min, 12% to 38% B; 65 to 72 min, 38 to 95% B; 72 to 80 min, 95% B; 80 to 81 min, 95 to 5% B; 81 to 95 min, 5% B.

The mass spectrometers used electrospray ionization (2 kV) at 275°C in “top speed” mode. Orbitrap resolution was 120,000, and for MS/MS it was 50,000. MS/MS spectra were acquired using a quadrupole isolation width of 1.6 *m/z* and higher-energy collisional dissociation (HCD) normalized collision energy (NCE) of 38. Dynamic exclusion was set for 30 s using monoisotopic precursor selection.

**(iv) Data processing.** Raw data files were searched using MSFragger 3.11 and Philosopher 3.3.11 against the Sus scrofa protein database from NCBI database (GCF_000003025.6_Sscrofa11.1). Mass tolerances for precursor and fragment ions were 10 ppm and 0.02 Da, respectively. Proteins and peptides were filtered using a false discovery rate (FDR) of <1%. The enzyme parameter was limited to semitryptic peptides with a maximum miscleavage of 2. Carbamidomethyl (C) of the peptides was set as the fixed modification; oxidation (M) and deamidated (NQ) on the N terminus of proteins were set as variable modifications. The PSM report from Philosopher to R used “PDtoMSstatsTMTFormat()” from the MSstatsTMT package to perform filtering on reporter ion intensities.

Totally, proteomics analysis was performed using ileal samples of 5 piglets at each time point. In total, 8,657 ileal proteins were identified, with 1,976 known proteins. The latter were clustered into 5 groups, and their functions were enriched by Metascape online.

### Ileal mucosa microbiota sequencing (24).

**(i) DNA extraction.** Total genomic DNA of ileal mucosa was isolated using an E.Z.N.A. stool DNA kit (Omega Bio-tek Inc., USA) following the manufacturer’s instructions. DNA quantity and quality were analyzed using NanoDrop 2000 (Thermo Scientific, USA) and 1% agarose gel.

**(ii) Library preparation and sequencing.** The V3-V4 region of the 16S rRNA gene was amplified using the primers 338F (5′-ACTCCTACGGGAGGCAGCAG-3′) and 806R (5′-GGACTACHVGGGTWTCTAAT-3′) with Barcode. The PCRs (total 30 μl) included 15 μl PhusionR high-fidelity PCR master mix (New England Biolabs), 0.2 mM primers, and 10 ng DNA. The thermal cycle was carried out with an initial denaturation at 98°C, followed by 30 cycles of 98°C for 10 s, 50°C for 30 s, and 72°C for 30 s, and a final extension at 72°C for 5 min. PCR products were purified using a AxyPrep DNA gel extraction kit (Axygen Biosciences, USA). The sequencing libraries were constructed with a NEBNext Ultra DNA library preparation kit for Illumina (New England Biolabs [NEB], USA) following the manufacturer’s instructions, and index codes were added. Then, the library was sequenced on the Illumina MiSeq 2500 platform (Illumina, USA), and 300-bp paired-end reads were generated at the Novo gene. The paired-end reads were merged using FLASH (V1.2.71). The quality of the tags was controlled in QIIME (V1.7.02); meanwhile, all chimeras were removed. The core set of the Greengenes database3 was used for classification, and sequences with >97% similarity were assigned to the same operational taxonomic units (OTUs).

**(iii) Analysis of sequencing data.** OTU abundance information was normalized using a standard of sequence number corresponding to the sample with the fewest sequences. The alpha diversity indices were calculated with QIIME (version 1.7.0). Partial least squares discrimination analysis (PLS-DA) was performed using R software (version 2.15.3).

### Histopathology analysis.

Segments of small intestinal tissue were fixed in 10% neutral formalin; they were subsequently paraffin embedded, cut into 5-μm sections, and stained with hematoxylin and eosin (H&E) for histopathological analysis.

### Immunofluorescent staining (IHF).

The protocol for immunofluorescence staining is reported in our recent publications (8, 24). Table S1 lists the primary antibodies that were used. Briefly, 5 μm thick tissue sections were cut and subjected to antigen retrieval. Sections were initially blocked with normal goat serum in PBS, followed by incubation (1:150 in PBS–1% BSA) with primary antibodies (Abs) overnight at 4°C. Following a brief wash, sections were incubated with goat anti-rabbit or donkey anti-goat secondary Abs (1:100 in PBS; Beyotime Institute of Biotechnology, Shanghai, People’s Republic of China) at RT for 30 min and finally counterstained with 4′,6-diamidino-2-phenylindole (DAPI). The stained sections were examined under a Nikon Eclipse TE2000-U fluorescence microscope (Nikon, Inc., Melville, NY), and the captured fluorescent images were analyzed using MetaMorph software.

10.1128/mSystems.00725-21.6 TABLE S1 Primary antibody information. Download Table S1, DOCX file, 0.02 MB. Copyright © 2021 Meng et al. 2021 Meng et al. https://creativecommons.org/licenses/by/4.0/ This content is distributed under the terms of the Creative Commons Attribution 4.0 International license.

### Western blotting.

Western blotting followed our previously reported protocols (8, 24). Briefly, small intestine tissue samples were lysed in radioimmunoprecipitation assay (RIPA) buffer containing the protease inhibitor cocktail from Sangong Biotech, Ltd. (Shanghai, China). Protein concentration was determined using a BCA kit (Beyotime Institute of Biotechnology). Information for primary antibodies is given in Table S1. Secondary donkey anti-goat Ab (catalog no. A0181) was purchased from Beyotime Institute of Biotechnology, and goat anti-rabbit (catalog no. A24531) Abs were purchased from Novex by Life Technologies (USA). Protein samples (50 μg/sample) were loaded onto 10% SDS-polyacrylamide electrophoresis gels. The gels were transferred to a polyvinylidene fluoride (PVDF) membrane at 300 mA for 2.5 h at 4°C. Membranes were then blocked with 5% BSA for 1 h at RT, followed by three washes with 0.1% Tween 20 in TBS (TBST). The membranes were incubated with primary Abs diluted with 1:500 in TBST with 1% BSA overnight at 4°C. After a further three washes with TBST, the blots were incubated with horseradish peroxidase (HRP)-labeled secondary goat anti-rabbit or donkey anti-goat Ab, respectively, for 1 h at RT. Following a further three washes, the blots were imaged. Secondary donkey anti-goat Ab (catalog no. A0181) was purchased from Beyotime Institute of Biotechnology, and goat anti-rabbit Abs (catalog no. A24531) were purchased from Novex by Life Technologies.

### Statistical analysis.

For ileal mucosa microbiota data analysis, data that were not normally distributed following log transformation or that had unequal variances were subjected to nonparametric analysis using the Kruskal-Wallis test within the NPAR1WAY procedure of SAS.

### Data availability.

The 10× sequencing raw data have been deposited in NCBI’s Gene Expression Omnibus under accession number GSE162287. The proteomics data have been deposited at the Integrated Proteome resources (https://www.iprox.org/) with the ID IPX0002622001. The microbiota raw sequencing data generated in this study have been uploaded to the NCBI SRA database with the accession number PRJNA681460.
