# Supplementary figures and images for: Single-Cell Transcriptome Sequencing and Proteomics Reveal Neonatal Ileum Dynamic Developmental Potentials
Source: mSystems. 2021 Sep 21;6(5):e00725-21. doi: 10.1128/mSystems.00725-21 (PMC8547457; doi:10.1128/mSystems.00725-21)

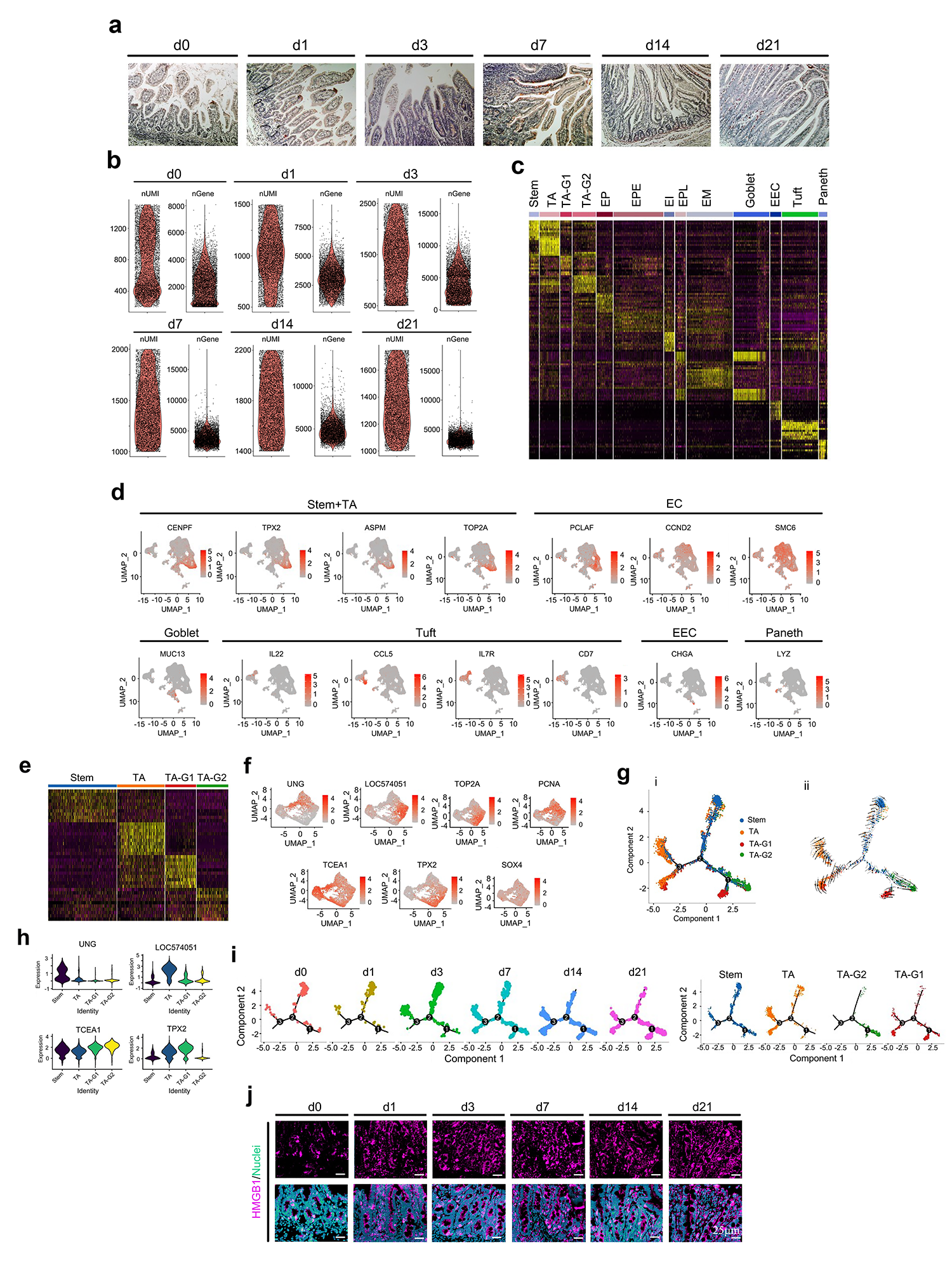

Supplement: FIG S1 [file msystems.00725-21-sf001.tif]

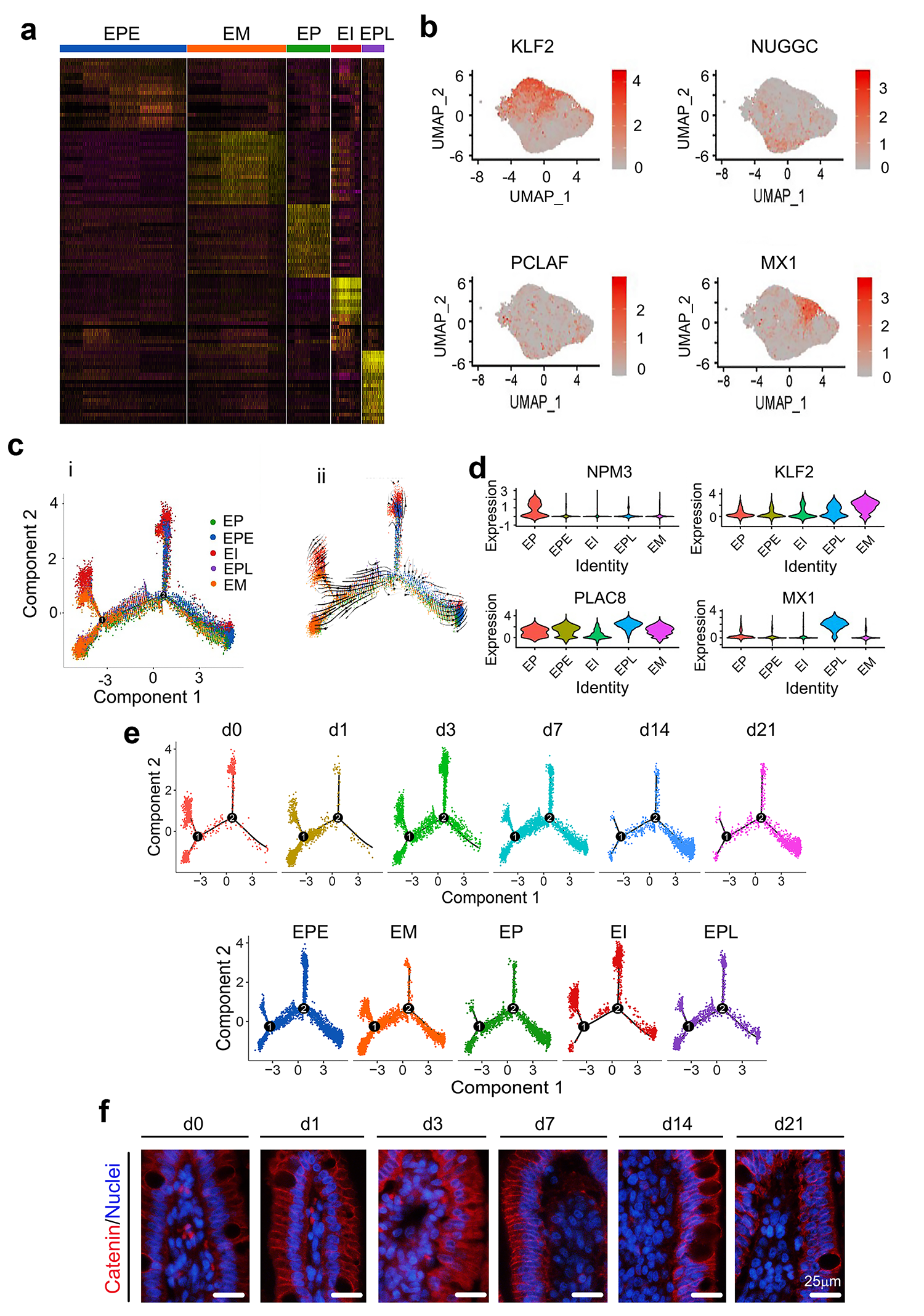

Supplement: FIG S2 [file msystems.00725-21-sf002.tif]

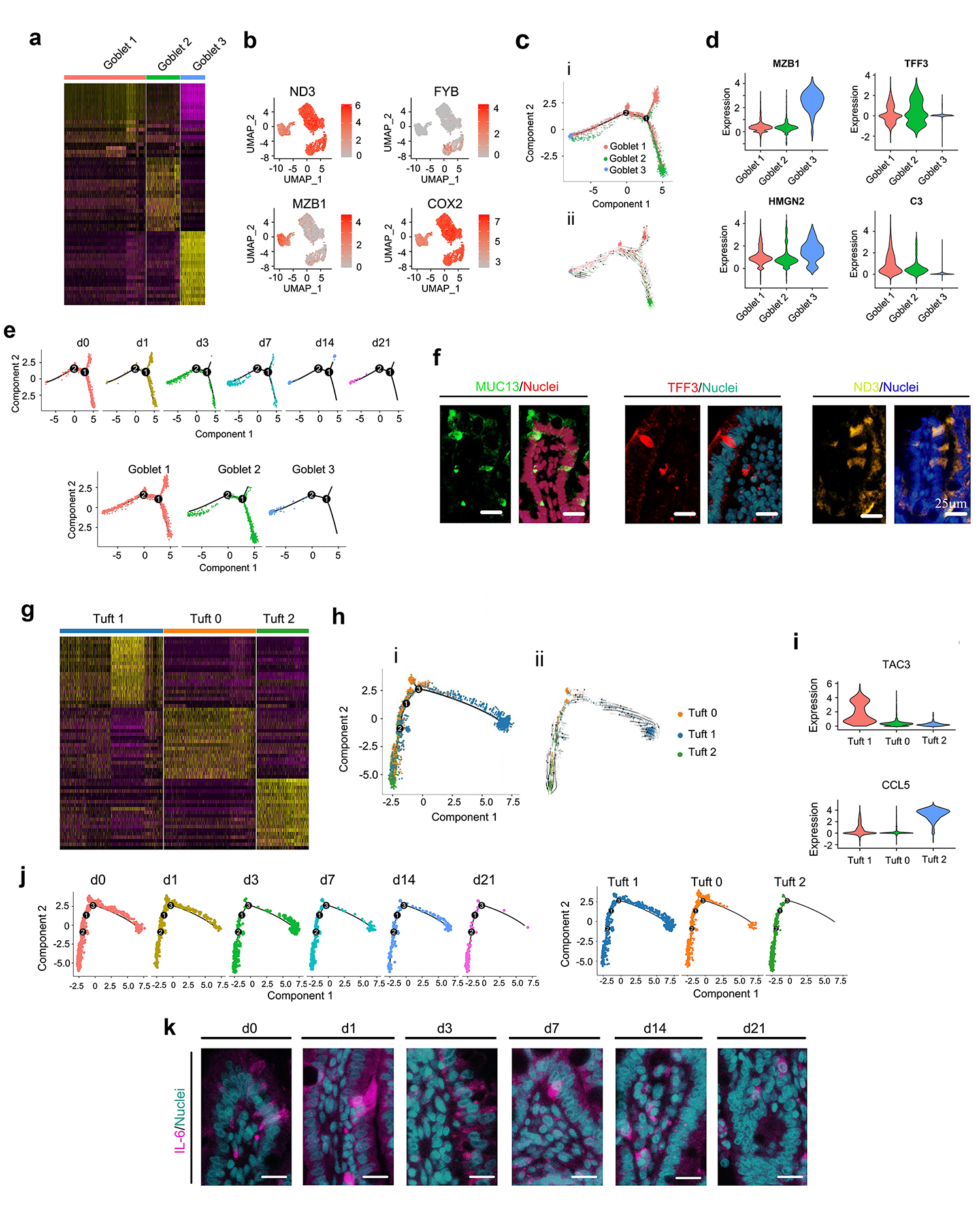

Supplement: FIG S3 [file msystems.00725-21-sf003.tif]

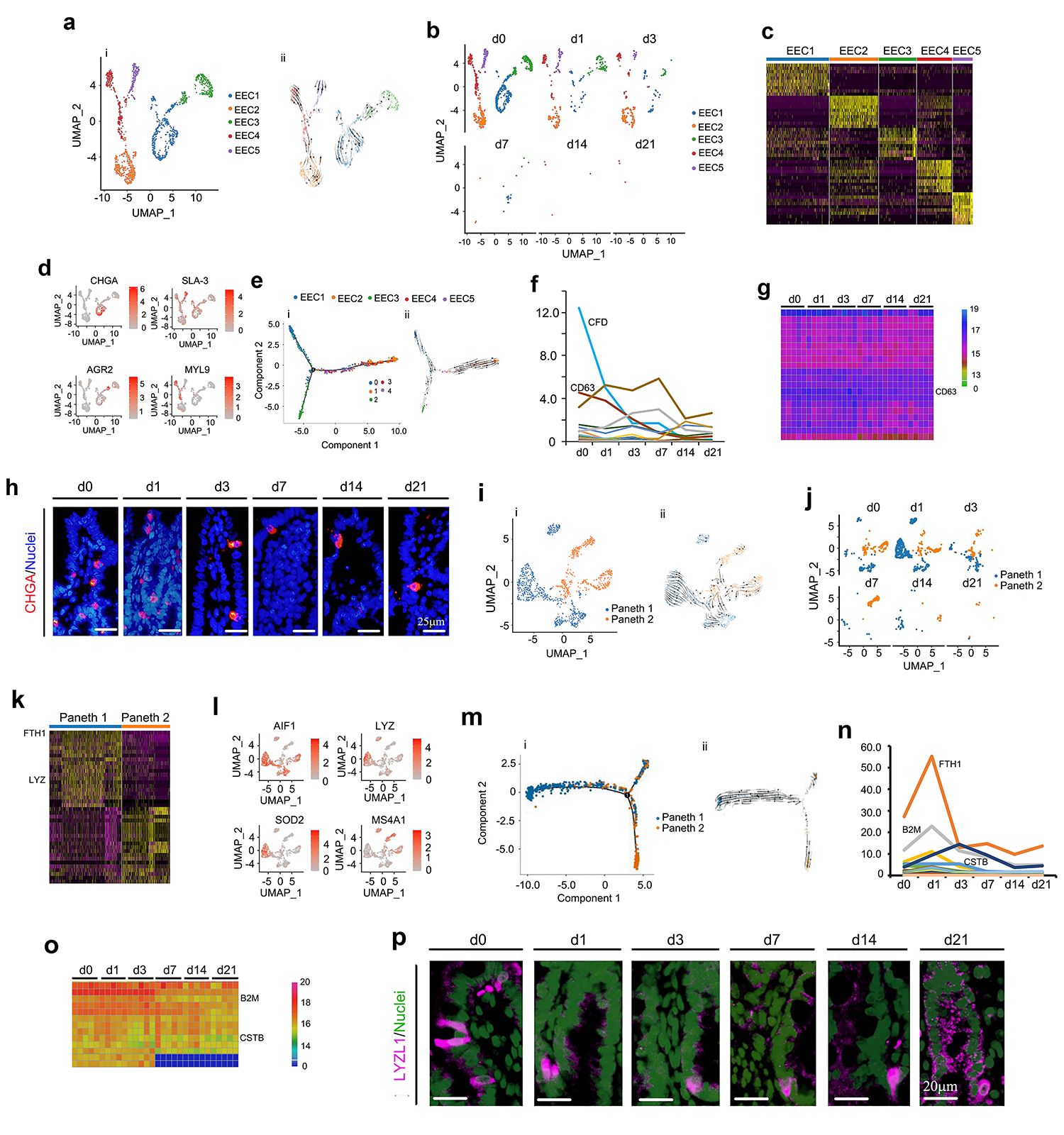

Supplement: FIG S4 [file msystems.00725-21-sf004.tif]

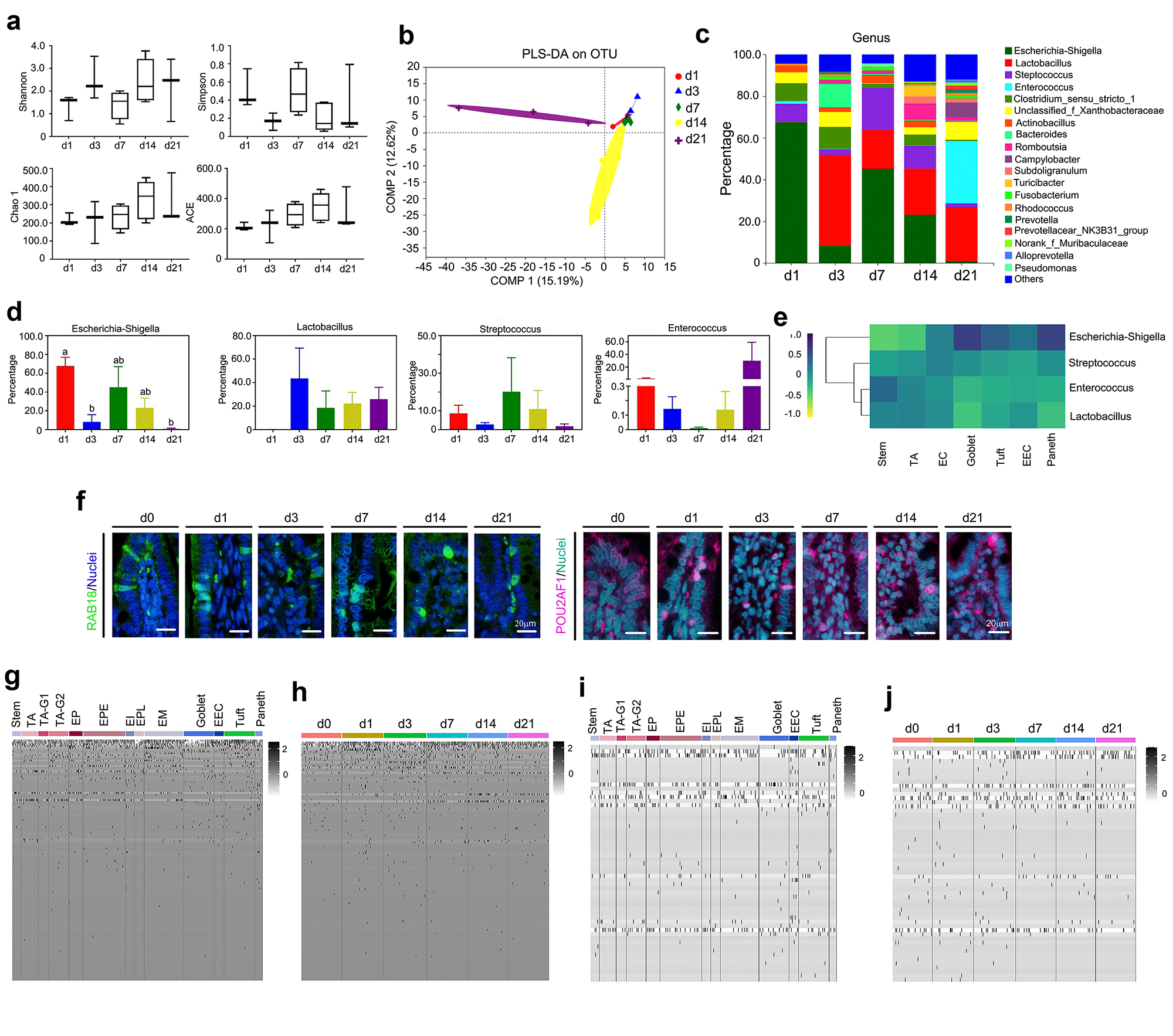

Supplement: FIG S5 [file msystems.00725-21-sf005.tif]
