# Supplementary material for: Single-Cell Transcriptome Sequencing and Proteomics Reveal Neonatal Ileum Dynamic Developmental Potentials
Source: mSystems. 2021 Sep 21;6(5):e00725-21. doi: 10.1128/mSystems.00725-21 (PMC8547457; doi:10.1128/mSystems.00725-21)
Supplement: TABLE S1 [file msystems.00725-21-st001.docx]

**Supplementary Table 1.** Primary antibodies

| **Gene symbol** | **Name** | **Cat. #** | **Predicted size** | **Source (Animal)** | **Company** |
| --- | --- | --- | --- | --- | --- |
| Actin | Actin | D110001-0200 | 42kd | Rabbit (polyclonal) | BBI |
| PCNA | Rabbit Anti-PCNA antibody | ab18197 | 29kd | Rabbit (polyclonal) | Abcam |
| SOX9 | Anti-Sox9 Antibody | AB5535 | 65kd | Rabbit (polyclonal) | Merck Millipore |
| CD74 | Rabbit Anti-CD74 antibody | bs-2518R | 34kd | Rabbit (polyclonal) | Beijing Biosynthesis Biotechnology CO. |
| FABP | Rabbit Anti-H-FABP antibody | ab45966 | 15kd | Rabbit (polyclonal) | Abcam |
| COX2 | Rabbit Anti-COX2/Cyclooxygenase 2 antibody | bsm-52502R | 65kd | Rabbit (monoclonal) | Beijing Biosynthesis Biotechnology CO. |
| CCL5 | Rabbit Anti-CCL5/RANTES antibody | bs-20765R | 7.4/10kDa | Rabbit (polyclonal) | Beijing Biosynthesis Biotechnology CO. |
| E2F8 | Rabbit Anti-E2F8 antibody | bs-4265R | 94kd | Rabbit (polyclonal) | Beijing Biosynthesis Biotechnology CO. |
| CREM | Rabbit Anti-CREM antibody | D152356 | 39kd | Rabbit (polyclonal) | BBI |
| PAX5 | Rabbit Anti-PAX5 antibody | bs-1861R | 42kd | Rabbit (polyclonal) | Beijing Biosynthesis Biotechnology CO. |
| EGR1 | Rabbit Anti-Egr1 antibody | bs-1076R | 60kd | Rabbit (polyclonal) | Beijing Biosynthesis Biotechnology CO. |
| HMGB1 | Rabbit Anti-HMGB1 antibody | bs-0664R | 25kd | Rabbit (polyclonal) | Beijing Biosynthesis Biotechnology CO. |
| Catenin | Rabbit Anti-delta 1 Catenin/CAS antibody | ab92514 | 108kd | Rabbit (monoclonal) | Abcam |
| MUC13 | Mouse Anti-MUC13 antibody | ab231159 | 55kd | Mouse (monoclonal) | Abcam |
| TFF3 | Rabbit Anti-Trefoil Factor 3 antibody | ab202967 | 9kd | Rabbit (polyclonal) | Abcam. |
| ND3 | Rabbit Anti-MT-ND3 antibody | ab192306 | 13kd | Rabbit (polyclonal) | Abcam |
| IL-6 | Rabbit Anti-IL-6 antibody | bs-0782R | 23kd | Rabbit (polyclonal) | Beijing Biosynthesis Biotechnology CO. |
| CHGA | Rabbit Anti-Chromogranin A antibody | ab45179 | 86kd | Rabbit (polyclonal) | Abcam |
| LYZL1 | Rabbit Anti-LYZL1 antibody | bs-18593R | 15kd | Rabbit (polyclonal) | Beijing Biosynthesis Biotechnology CO. |
| RAB18 | Mouse Anti-RAB18 antibody | bsm-51333M | 23kd | Mouse (monoclonal) | Beijing Biosynthesis Biotechnology CO. |
| POU2AF1 (BOB1) | Rabbit Anti-BOB1 antibody | bs-1418R | 28kd | Rabbit (polyclonal) | Beijing Biosynthesis Biotechnology CO. |
